# Pharmacological potential of tocotrienols: a review

**DOI:** 10.1186/1743-7075-11-52

**Published:** 2014-11-12

**Authors:** Haseeb Ahsan, Amjid Ahad, Jahangir Iqbal, Waseem A Siddiqui

**Affiliations:** Department of Biochemistry, Faculty of Dentistry, Jamia Millia Islamia, New Delhi, 110025 India; Department of Biochemistry, Jamia Hamdard (Hamdard University), New Delhi, 110062 India; Department of Cell Biology and Pediatrics, SUNY Downstate Medical Center, Brooklyn, NY 11203 USA

**Keywords:** Dietary tocotrienols, Pharmacology, Antioxidant, Anti-inflammatory, Hypolipidemic, Hypoglycaemic, Anti-cancer, Cardioprotective

## Abstract

Tocotrienols, members of the vitamin E family, are natural compounds found in a number of vegetable oils, wheat germ, barley, and certain types of nuts and grains. Like tocopherols, tocotrienols are also of four types viz. alpha, beta, gamma and delta. Unlike tocopherols, tocotrienols are unsaturated and possess an isoprenoid side chain. Tocopherols are lipophilic in nature and are found in association with lipoproteins, fat deposits and cellular membranes and protect the polyunsaturated fatty acids from peroxidation reactions. The unsaturated chain of tocotrienol allows an efficient penetration into tissues that have saturated fatty layers such as the brain and liver. Recent mechanistic studies indicate that other forms of vitamin E, such as γ-tocopherol, δ-tocopherol, and γ-tocotrienol, have unique antioxidant and anti-inflammatory properties that are superior to those of α-tocopherol against chronic diseases. These forms scavenge reactive nitrogen species, inhibit cyclooxygenase- and 5-lipoxygenase-catalyzed eicosanoids and suppress proinflammatory signalling, such as NF-κB and STAT. The animal and human studies show tocotrienols may be useful against inflammation-associated diseases. Many of the functions of tocotrienols are related to its antioxidant properties and its varied effects are due to it behaving as a signalling molecule. Tocotrienols exhibit biological activities that are also exhibited by tocopherols, such as neuroprotective, anti-cancer, anti-inflammatory and cholesterol lowering properties. Hence, effort has been made to compile the different functions and properties of tocotrienols in experimental model systems and humans. This article constitutes an in-depth review of the pharmacology, metabolism, toxicology and biosafety aspects of tocotrienols. Tocotrienols are detectable at appreciable levels in the plasma after supplementations. However, there is inadequate data on the plasma concentrations of tocotrienols that are sufficient to demonstrate significant physiological effect and biodistribution studies show their accumulation in vital organs of the body. Considering the wide range of benefits that tocotrienols possesses against some common human ailments and having a promising potential, the experimental analysis accounts for about a small fraction of all vitamin E research. The current state of knowledge deserves further investigation into this lesser known form of vitamin E.

**Electronic supplementary material:**

The online version of this article (doi:10.1186/1743-7075-11-52) contains supplementary material, which is available to authorized users.

## Introduction

Evans and Bishop, in 1922, discovered that dietary supplements with alfalfa leaves (rich in vitamin E) prevent placental hemorrhage and reverse dietary sterility in rats [1]. Evans and his associates [2] isolated the compounds of vitamin E family and named them tocopherols (Greek: Tocos-child birth; pheros- to bear; ol-alcohol). While alpha-tocopherol was the first vitamin E isomer to be recognized, eight chemically distinct isomers are now known, consisting of alpha (α), beta (β), gamma (γ) and delta (δ)-tocopherols and α, β, γ and δ-tocotrienols (T3), all of them are referred to as vitamin E. The tocopherols are saturated forms of vitamin E, whereas the tocotrienols are unsaturated and possess an isoprenoid side chain (Table 1). The name “tocotrienol” was first suggested by Dr. Banyan, for the isomers of vitamin E, with isoprenoid side chain present in nature, when isolated from the latex of the rubber plant, *Havea brasiliensis*[3]. Tocotrienols attracted no real attention until the 1980’s and 1990’s when their cholesterol-lowering potential [4] and anticancer effects were described [5, 6]. This review article will take a closer look at the various functions of tocotrienol by providing numerous potential evidences on how it may be protective against these chronic diseases. Tocotrienols are found in certain cereals and vegetables such as palm oil, rice bran oil, coconut oil, barley germ, wheat germ and annatto [7, 8]. Palm oil and rice bran oil contain particularly higher amounts of tocotrienols (940 mg/kg and 465 mg/kg, respectively) [9]. Other sources of tocotrienols include grape fruit seed oil, oats, hazelnuts, maize, olive oil, Buckthorn berry, rye, flax seed oil, poppy seed oil and sunflower oil.

**Table 1:** **Structures of various homologs of tocotrienols**

Type	R1	R2	R3	Structure
*alpha(* α*)*-Tocotrienol	Me	Me	Me	
*beta(*β*)*-Tocotrienol	Me	H	Me	
*gamma(*γ*)*-Tocotrienol)	H	Me	Me	
*delta(*δ*)*-Tocotrienol)	H	H	Me	

Tocotrienols possess powerful neuroprotective, antioxidant, anti-cancer and cholesterol lowering properties that often differ from the properties of tocopherols [10]. Micromolar amounts of tocotrienol suppress the activity of HMG-CoA reductase, the hepatic enzyme responsible for the synthesis of cholesterol [11, 12]. Tocotrienols are thought to have more potent antioxidant properties than α-tocopherol [13, 14]. The unsaturated side chain of tocotrienol allows for more efficient penetration into tissues that have saturated fatty layers such as the brain and liver [15]. Experimental research examining the antioxidant, free radical scavenging, effects of tocopherol and tocotrienols have found that tocotrienols appear superior due to their better distribution in the lipid layers of the cell membrane [16]. One major conclusion often used to undermine tocotrienol research is the relative inferiority of the bioavailability of orally taken tocotrienols as compared to that of α-tocopherol. The hepatic α-tocopherol transfer protein (α-TTP), together with the tocopherol-associated proteins (TAP) is responsible for the endogenous accumulation of natural α-tocopherol.

Tocotrienols are absorbed, in the same way as other vitamin E compounds, alongwith fat, in the small intestine, after being cleaved by the enzyme esterase, located in the stomach lining. Bile salts are necessary for the absorption. It is then packaged into chylomicrons and then transported in the lymphatic system. The α-tocotrienol appears to be better absorbed than the other forms of tocotrienol. In the bloodstream, tocotrienols are exposed to the oxidative free radicals and therefore perform most of their antioxidant activity. Tissue uptake takes place either with the help of lipoprotein lipases, digesting the lipoprotein constituents, or by receptor mediated endocytosis of lipoprotein. Lipoprotein lipase degrades lipoproteins to remnant particles which are then taken up by liver or peripheral tissues by receptor mediated endocytosis. Tocotrienols enter a variety of different tissue types, with adipose and adrenal gland having the highest levels. Vitamins can be stored in the tissue for long periods of time because of their exceedingly slow turnover rate. Vitamin E is oxidized after it has performed its antioxidant function. It is converted to its hydroquinone form in a P450 dependent manner before being eliminated from the body through faeces. Hydroquinone form binds with glucuronic acid and mixes with bile for removal through faeces. Despite the promising potential of tocotrienol, the experimental analysis accounts for only a small fraction of all vitamin E research. However, biologists are increasingly realizing the importance of this minor and unique vitamin E isomer [16].

Various minerals and vitamins are present in a variety of food products and available as dietary supplements. Selenium (Se) is an essential micronutrient that occurs predominantly as selenomethionine (SeMet), whereas vitamin E (or a-tocopherol) is a fat-soluble physiological antioxidant, both of which are required for normal health [17–19]. The Se and vitamin E are essential components of the human diet and have been studied as antioxidants and potential therapeutic agents for a variety of human diseases. Various formulations of Se and vitamin E have been shown to possess a therapeutic and preventive effect against prostate cancer (PCa) cells [20].

The motivation for the use of vitamin E and Se for the prevention of PCa comes from clinical trial data. Vitamin E was shown to be a promising candidate for PCa prevention in the α-Tocopherol β-Carotene Cancer Prevention Study, a controlled smoking trial where α-tocopherol reduced PCa incidence by 32% and mortality by 41% [21]. The SUpplementation en VItamines et Mineraux AntioXidants (SUVIMAX) study found a significant reduction in PCa rates among men receiving a multivitamin containing 30 mg vitamin E, although the protective effect could not be attributed to any specific micronutrient [22]. In contrast, the Heart Outcomes Prevention Evaluation (HOPE) trial, the Heart Protection Study, the NIH-AARP Diet and Health Study and the Cancer Prevention Study II Nutrition Cohort donot support a general protective effect of α-tocopherol supplement use for PCa prevention [23–26].

Therefore, the Selenium and Vitamin E Cancer Prevention Trial (SELECT), was designed to test a prostate cancer chemoprevention hypothesis using oral Se and vitamin E supplementation in disease-free volunteers. Initiated in 2001, the SELECT was a phase III, randomized, placebo-controlled human trial to investigate the PCa chemopreventive effects of Se, vitamin E or their combination [27]. SELECT was among the largest clinical chemoprevention trials ever, with an enrollment of more than 35,000 men and an intended follow-up of up to 12 years [27]. SELECT was predicated on basic and clinical research including secondary endpoint data from cancer prevention studies that implied Se and vitamin E supplements could be useful in reducing PCa risk. However, the trial was prematurely terminated in 2008, 18 months before its intended minimum follow-up length. The Se and vitamin E doses and formulations used in SELECT were found to be ineffective, and concern was raised about a possible trend in developing type 2 diabetes mellitus among the study participants taking Se [27]. Further, a statistically nonsignificant increased risk of PCa was also seen in the vitamin E group participants. Unfortunately, despite the perceived suitability of PCa for chemoprevention and the considerable evidence suggesting the usefulness of Se and vitamin E for PCa prevention, SELECT failed to show a positive effect. Hence, the SELECT trial was terminated early because of the safety concerns and negative data for the formulations and doses given [28].

The biological activity of vitamin E has generally been associated with its well-defined antioxidant property, specifically against lipid peroxidation in biological membranes. In the vitamin E group, a-tocopherol is considered to be the most active form. Moreover, tocotrienol has been shown to possess novel hypocholesterolemic effects together with an ability to reduce the atherogenic apolipoprotein B and lipoprotein(a) plasma levels. In addition, tocotrienol has been suggested to have an anti-thrombotic and anti-tumor effect indicating that tocotrienol may serve as an effective agent in the prevention and/or treatment of cardiovascular disease and cancer. The physiological activity of tocotrienol suggests it to be superior than a-tocopherol in many pathophysiological conditions. Hence, the role of tocotrienol in the prevention of cardiovascular disease and cancer may have significant clinical implications. Additional studies on its mechanism of action, as well as, long-term intervention studies from the pharmacological point-of-view are required to elucidate its function [29].

## Biochemical functions

Vitamin E is not a single agent but is at least eight “vitamers,” named tocochromanols and can be either “tocopherols” or “tocotrienols”. They are further assigned Greek letter prefixes by degree of methyl substitution, that is, α-tocopherol (with three chiral centers) exists naturally as the (2R,4′R,8′R) stereoisomer and is synthesized as all-racemic or (2RS,4′RS,8′RS) product [30]. In 1943, Joffe and Harris demonstrated varying potencies of the eight forms of vitamins [31]. It is known that α-tocopherol is taken up in the human liver by hepatic transfer proteins and some tocopherol stereoisomers penetrate cell membranes more easily [31, 32]. The 2R isomers of α-tocopherol are putatively used to establish the vitamin E requirement [33]. As commercial dietary supplements, both natural (RRR) and synthetic (all-rac) α-tocopheryl esters (acetate or succinate) are available and extensively used. Lee *et al*. [34] showed that vitamin E succinate has a distinct anti-prostaglandin effect in human lung epithelial cells. Vitamin E is relatively safe for consumption even at high dosages, since a 3,200-IU dose was well tolerated by adults in short-term studies [35].

One of the most significant differences between αT and other forms of vitamin E is that in contrast to αT, which is mostly retained in tissues because of preferential binding by α-TTP, large quantities of other forms of vitamin E are readily metabolized by CYP4F2-initiated ω-oxidation of the side chain to generate carboxychromanols and conjugated counterparts. Short-chain carboxychromanols such as CEHCs are excreted in the urine and γ-CEHC has been shown to have natriuretic activities [36]. Long-chain carboxychromanols, especially 13′-COOHs, are found in tissues and faeces in animals supplemented with γT, δT, and γTE [37–40]. The discovery of potent anti-inflammatory [41, 42] and anticancer [43] effects of long-chain carboxychromanols represents an exciting research direction and provides new insights into the physiological roles of less tissue-preserved forms of vitamin E [44].

α-tocopherol, the commonly studied isomer of vitamin E, has been shown to possess an anti-cancer effect [45–47]. However, the researchers have also started evaluating γ-tocopherol for its chemopreventive efficacy [45–51]. Interestingly, γ-tocopherol is being increasingly appreciated to have a better potential than α-tocopherol [52]. Studies have shown that the *in vitro* products of γ-tocopherol antioxidant reactions differ from those of α-tocopherol, with the latter alone forming nitrosating agents when exposed to NO_2_[52]. Christen *et al*. [53] showed that γ-tocopherol may be required to complement the activity of α-tocopherol, suggesting that supplementation of α-tocopherol alone may suppress γ-tocopherol levels. The majority of available experimental data on vitamin E for suppressing PCa has been obtained with α-tocopherol, leaving the possibility that other vitamin E isomers may possess better chemopreventive or anticancer potential against PCa, but which needs to be explored further [28].

All tocopherols and tocotrienols are potent antioxidants with lipoperoxyl radical-scavenging activities. Only until recently, most research on vitamin E has primarily focused on αT [54], because αT is the predominant form of vitamin E in tissues and low intake of this form results in vitamin E deficiency-associated ataxia [55]. However, many human and animal studies on αT supplementation have yielded disappointing outcomes regarding its protective role in prevention or treatment of chronic diseases including cardiovascular diseases and cancer [56, 57]. On the other hand, recent mechanistic studies combined with preclinical animal models have indicated that compared with αT, other forms of vitamin E appear to have different and superior biological properties that may be useful for prevention and therapy against chronic diseases. Furthermore, emerging evidence suggests that some long-chain vitamin E metabolites have even stronger anti-inflammatory effects than their vitamin precursors. These metabolites may be novel anti-inflammatory agents and may contribute to beneficial effects of vitamin E forms in vivo. Here we discuss recent developments in the field of non-αT forms of vitamin E with respect to their metabolism and antioxidant and anti-inflammatory effects [44].

Most of the functions of tocotrienols are related to its antioxidant property in animals. It prevents the non-enzymatic oxidations of various cell components (e.g. unsaturated fatty acids) by molecular oxygen and free radicals such as superoxide (O_2_^−^) and hydrogen peroxide (H_2_O_2_). The various biochemical functions of tocotrienols are related either directly or indirectly to its antioxidant property. They are essential for membrane structure and integrity of the cell [58]. Tocotrienols prevent the peroxidation of polyunsaturated fatty acids in various tissues and membranes and protects the red blood cells from hemolysis by oxidizing agents [59]. It increases the synthesis of heme by enhancing the activity of enzymes δ-aminolevulinic acid (ALA) synthase and ALA dehydratase. It is required for cellular respiration through electron transport chain and is believed to stabilize coenzyme Q. It prevents the oxidation of vitamin A and carotenes and also LDL and thus may be helpful in the prevention of some chronic diseases [9, 59]. Tocotrienols also protects the liver from being damaged by toxic compounds such as CCL_4_.

## Pharmacological properties

Tocotrienols have a very broad range of medicinal properties and are used as antioxidant, analgesic, anti-inflammatory, antibacterial, antipyretic, antithrombotic, anticancer, cardioprotective, hepatoprotective, hypoglycemic, and nephroprotective, as discussed below. The pharmacological potential of tocotrienols has also been summarized in Table 2.

**Table 2:** **Biological properties of tocotrienols**

S.No.	Protective activity	Tocotrienol type	Proposed mechanism of action	References
1.	Anti-cancer	γ-T_3_	Inhibition of NF-κB, TGF-β and P38 signalling pathways	[8, 143]
		γ-T_3_, δ-T_3_	Induction and potentiation of apoptosis	[20, 22, 31, 144, 145]
		α-T_3_, γ-T_3_, δ-T_3_	Activation of caspases	[31, 34, 61, 145, 146]
		γ-T_3_, δ-T_3_	Down-regulation of Bcl-2 and cyclin D	[61]
		α-T_3_, γ-T_3_	Suppression of HMGR activity	[44]
		TRF from palm oil	Induction of DNA fragmentation	[18]
		α-T_3_, δ-T_3_	Inhibition of angiogenesis	[55]
		γ-T_3_, δ-T_3_	Inhibition of cell proliferation through cell cycle arrest	[25, 33]
		γ-T_3_, δ-T_3_	Down-regulation of Raf/Erk pathway	[27]
2.	Anti-diabetic	TRF from palm oil and rice bran oil	Prevents the formation of advanced glycationendproducts in diabetic rats	[72]
		α-T_3_, γ-T_3_, δ-T_3_	Reduces hyperglycemia and hyperlipidemia in diabetic rats	[73]
		α-T_3_, γ-T_3_, δ-T_3_	Inhibition of NF-κB signalling pathway	[75]
		α-T_3_, γ-T_3_, δ-T_3_	Inhibition of oxidative-nitrosative stress	[120]
		α-T_3_, γ-T_3_, δ-T_3_	Inhibition of TNF-α, IL-1β, TGF-β1 and caspase-3 activity	[74, 77]
		TRF from palm oil and rice bran oil	Reduction of glucose-insulin index	[79, 80]
		α-T_3_, γ-T_3_, δ-T_3_	Increase in insulin sensitivity	[59, 81, 83]
3.	Anti-inflammatory	α-T_3_, γ-T_3_, δ-T_3_	Suppression of NF-κB, TNF-α, IL-1, IL-6, IL-8 and iNOS	[50, 56, 74, 147]
		α-T_3_, γ-T_3_, δ-T_3_	Suppression of cyclooxygenase-2 activity	[51, 57]
		α-T_3_, γ-T_3_, δ-T_3_	Suppression of STAT-3 signalling pathway	[29, 45]
4.	Antioxidant	α-T_3_, γ-T_3_, δ-T_3_	Increase in the activity of antioxidant enzymes	[59, 60, 62, 65, 148]
		TRF from palm oil and rice bran oil, α-T_3_, γ-T_3_, δ-T_3_	Quenching and scavenging of free radicals	[63, 69, 70, 79]
		α-T_3_, γ-T_3_, δ-T_3_	Inhibition of lipid peroxidation	[64, 66, 68]
5.	Immuno-stimulatory	α-T_3_, δ-T_3_	Induction of antibody production	[99, 101]
		α-T_3_, γ-T_3_, δ-T_3_	Induction of IFN-γ, IL-4, IL-1β production	[99, 102]
		δ-T_3_	Suppression of TNF-α	[102]
6.	Cardio-protective	α-T_3_, γ-T_3_	Inhibition of HMG-CoA reductase activity	[10, 86, 104]
		α-T_3_, γ-T_3_	Inhibition of expression of cell adhesion molecules	[105]
		α-T_3_, γ-T_3_	Reduction in the levels of blood cholesterol	[106, 107]
		TRF from palm oil and rice bran oil, δ-T_3_	Inhibition of lipid peroxidation	[41, 80]
		γ-T_3_, δ-T_3_	Downregulation of c-Src expression	[102]
		γ-T_3_, δ-T_3_	Upregulation of phosphorylation of Akt	[102]
		TRF from palm oil	Reduction in the production of apolipoprotein B, platelet derived factor-4, thromboxane B2	[149]
		TRF from palm oil and rice bran oil	Downregulation of TGF-β	[80]
7.	Neuro-protective	α-T_3_	Inhibition of PP 60 (c-Src) kinase activity and phosphorylation of Erk	[112]
		α-T_3_, γ-T_3_	Inhibition of 12-lipoxygenase activity	[115, 116]
		α-T_3_, γ-T_3_, δ-T_3_	Reduction of oxidative stress	[77]
8.	Hepato-protective	α-T_3_, γ-T_3_	Inhibition of lipid peroxidation and oxidative damage	[62, 64, 65, 68]
		γ-T_3_, δ-T_3_	Induction of the expression of CYP450, UGT1A1 nad MDR-protein 1	[135, 136]
		TRF from palm oil and rice bran oil, α-T_3_, γ-T_3_, δ-T_3_	Induction of hepatic antioxidant status	[59, 137, 138]
9.	Nephro-protective	TRF from rice bran oil, α-T_3_, γ-T_3_	Inhibition of oxidative-nitrosative stress	[12, 136, 141]
		TRF from palm oil and rice bran oil, α-T_3_, γ-T_3_	Downregulating the expression of NF-κB, TGF-β, TNF-α and caspase-3	[75, 79, 80, 136, 139]

### Anti-cancer effects

The anticancer properties of tocotrienols are well known and documented [60–76]. Tocotrienols not only suppress cancer-cell proliferation, but also induces apoptosis in cancer cells. It has been reported that γ- and δ-tocotrienols exhibit greater anticancer activity than α- or β-tocotrienols [76–78]. The mechanism of anti-cancer effects of tocotrienols has been worked out [5, 79, 80]. They exert anti-cancer activity on cancer cells by cell cycle arrest through induction of cell cycle inhibitory protein and decreased expression of cyclin dependent kinase [64, 65, 79]. Tocotrienols also work as an anti-cancer agent by inhibiting angiogenesis [81, 82] or by enhancing immunity and inhibiting tumor cell migration [71]. Tocotrienol induces cell-cycle arrest and mitochondria-mediated apoptosis in human pancreatic cancer cells [68, 70, 75, 83]. It has also been shown to inhibit the tumor cell growth by suppressing HMG-CoA reductase activity [84]. It has been shown to induce apoptosis in stomach cancer cells through down-regulation of the Raf-ERK signaling pathway [69]. Tocotrienol significantly activated caspase-dependent programmed cell death in skin and pancreatic cells [73, 76]. γ- and δ-tocotrienols derived from palm oil exhibited strong activity against tumor promotion by inhibiting Epstein–Barr virus (EBV) early antigen expression in EBV-genome-carrying human lymphoblastoid cells induced by phorbol ester [9]. Tocotrienols suppressed DMBA-induced mammary tumors and hypercholesterolemia in murine model [85]. It induced apoptosis in human fibroblast cells through TGF-beta–Fas–JNK-signaling pathways [47]. Gamma-Tocotrienol induced poly (ADP-ribose) polymerase (PARP) cleavage and stimulated a rise in caspase-3, caspase-8 and caspase-9 activities in human hepatoma Hep3B cells [68]. The antiproliferative activity of tocotrienols are mediated through modulation of growth factors such as vascular endothelial growth factor (VEGF) [82], basic fibroblast growth factor (bFGF) [86] and transforming growth factor-beta (TGF-β) [62], HER2/neu [87], and interleukin-6 (IL-6) [88]. Cyclin-dependent kinases (CDK2, CDK4, CDK6) and their inhibitors, such as p21, p27 and p53 [60, 64] and downregulation of Rb phosphorylation [65, 66] also mediate the growth-suppressive effects of this agent. Downregulation of the telomerase, c-myc, and raf–ERK signaling pathways has been linked to tocotrienol’s ability to inhibit cell survival [69, 89].

Pancreatic cancer is a leading cause of cancer mortality with less than 5% of patients surviving 5 years after diagnosis [90]. Several studies have combined natural compounds that inhibit NF-kB, such as genistein, curcumin, fisetin, and green tea, to investigate synergy in treating pancreatic cancer [91–94]. However, translation of these studies to the clinic has been challenging due to the low bioavailability of some of these natural compounds in humans. Tocotrienols, a group of 4 (α-, β-, δ-, and γ-tocotrienol) unsaturated naturally occurring vitamin E compounds have received increasing attention for their potential as nontoxic dietary anticancer agents [95, 96]. Husain et al. [97] has shown that oral administration of 100 mg/kg/d of d-tocotrienol to mice resulted in levels that were 10 times higher in pancreas than in subcutaneously implanted tumor tissue, suggesting that these compounds will have reasonable bioavailability for pancreatic tumor intervention [97]. In another study, they investigated the potential of the natural tocotrienols to inhibit pancreatic cancer and NF-kB activation in vitro and in vivo. In addition, they also investigated the potential of the most bioactive tocotrienol to augment gemcitabine activity in vitro and in vivo [98]. Their results show that d- and g-tocotrienol inhibited NF-kB activity, cell growth, cell survival, and tumor growth in nude mice. It was shown by them that d-tocotrienol augmented gemcitabine activity in vitro and in vivo. The results suggest that inhibition of NF-kB signaling by d-tocotrienol may be an effective approach for the prevention and treatment of pancreatic cancer. Our findings suggest evaluation of NF-kB signaling compounds as an endpoint biomarker in the ongoing phase I trial of d-tocotrienol in patients with pancreatic tumors [98].

Breast cancer is the second most frequent cancer affecting women worldwide after lung cancer. The toxicity associated with chemical drugs has turned the attention toward natural compounds as anticancer agents. Vitamin E derivatives consisting of tocopherols and their analogs namely tocotrienols have been extensively studied due to their remarkable biological properties. While tocopherols have failed to offer protection, tocotrienols, in particular, a-, d-, and c-tocotrienols alone and in combination have demonstrated anticancer properties. The antiangiogenic, antiproliferative, and apoptotic effect of tocotrienols not only suggests that they are potent antitumor agents but also reinforces the notion that tocotrienols are indeed more than antioxidants [99].

Nesaretnam et al. [100] conducted a double-blinded, placebo-controlled pilot trial to test the effectiveness of adjuvant tocotrienol therapy in combination with tamoxifen for 5 years in women with early breast cancer. Two-hundred forty women, aged between 40 and 60 years, with either tumor node metastases (TNM) breast cancer and estrogen receptor (ER)-positive tumors were non-randomly assigned to two groups. The intervention group received tocotrienol-rich fraction (TRF) plus tamoxifen, whereas the control group received placebo plus tamoxifen, for 5 years. From the study, it was found that there is no association between adjuvant tocotrienol therapy and breast cancer-specific survival in women with early breast cancer. Hence, results from the study were not sufficient to indicate a significant association between adjuvant tocotrienol therapy and breast cancer survival in women with early breast cancer. However, evidence suggested that tocotrienols have anticancer effects and tamoxifen and tocotrienol *in vitro* demonstrate synergy. Although a 60% lower mortality occurred in the intervention group, this result was not statistically significant. Hence, a large randomized trial is certainly warranted in the near future to establish whether tocotrienol adjuvant therapy can significantly improve recurrence or mortality or both [100].

Nesaretnam et al. [99] have demonstrated a mechanism for tocotrienol activity that involves estrogen receptor (ER) signaling. In silico simulations and in vitro binding analyses indicate a high affinity of specific forms of tocotrienols for ERb, but not for ERa. Moreover, they found that specific tocotrienols increase ERb translocation into the nucleus which, in turn, activates the expression of estrogen responsive genes (MIC-1, EGR-1 and Cathepsin D) in breast cancer cells only expressing ERb cells (MDA-MB- 231) and in cells expressing both ER isoforms (MCF-7). The binding of specific tocotrienol forms to ERb is associated with the alteration of cell morphology, caspase-3 activation, DNA fragmentation, and apoptosis. Furthermore, some clinical trials seem to suggest that tocotrienols in combination may have the potential to extend breast cancer-specific survival.

Advances in chemopreventive approaches would be an immense breakthrough in lowering the mortality rate associated with breast cancer in women. Supplementation or treatment with palm tocotrienols has shown encouraging results mainly from in vitro and in vivo studies. Hence, tThe studies conducted by Nesaretnam et al. [99] demonstrated that tocotrienols have convincing potential in suppressing and inhibiting the growth of mammary tumor cells. Combined treatment with statins, celecoxib, and tamoxifen resulted in a significantly enhanced synergistic response compared with high doses of treatment with individual compounds. Interestingly, this effect was observed using lower doses of the anticancer agent in combination with tocotrienols, suggesting that the toxicity factor related to these drugs may be avoided. The recent clinical trial, even though is reported as a null study, displayed promising reduction in the risk and recurrence free survival in women with early breast cancer. Nevertheless, tocotrienols exhibit potential as anticancer agents to be used in combination treatment as well as to enhance therapeutic responsiveness in breast cancer patients [99].

### Anti-inflammatory activity

Tocotrienols have been extensively studied for their anti-inflammatory property and very promising scientific evidences have been brought up. The activation of the transcription factor NF-κB has been closely linked with inflammation [101, 102]. Tocotrienols have been shown to suppress the expression of TNF-α [101, 103], IL-1 [104], IL-6 [105], IL-8 [106], inducible nitric oxide synthase [107], and cyclo-oxygenase 2 [41, 103], all of which mediate inflammation. Tocotrienols have also been shown to suppress STAT3 cell-signaling pathway, also involved in inflammation [71, 85]. Hypoxia-induced factor-1 is another pathway that has been linked with inflammation and is modulated by tocotrienols [106]. Treatment of streptozotocin-induced diabetic rats with tocotrienols (25 mg/kg, 50 mg/kg and 100 mg/kg body weight) for 10 weeks, significantly prevented behavioral, biochemical and molecular changes associated with diabetes through suppression of activation of the NF-κB signaling pathway [103]. Non-toxic concentrations of tocotrienol attenuated the tumour necrosis factor-α (TNF)-induced nuclear transcription factor (NF-κB) activation in human chronic myeloid leukemia cells (KBM-5), which are the key steps in the development of inflammation [101].

### Anti-oxidant activity

It is now well established that generation of free radicals (O_2_^.-^, H_2_O_2_ and OH^−^) from the incomplete reduction of molecular oxygen during aerobic respiration is closely related to cellular damage. Regulation of the balance between production of reactive oxygen species (ROS) by cellular processes and its removal by antioxidant defense system maintains normal physiological processes. The antioxidant activities of tocotrienols are mediated through induction of antioxidant enzymes such as superoxide dismutase [108, 109], NADPH: quinoneoxidoreductase [110], and glutathione peroxidase [111], which quench free radicals such as superoxide radicals [112]. Effects of tocotrienols on antioxidant defense system in various animal models have been studied from time to time. lntragastric administration of tocotrienol for 30 days caused a significant elevation in different components of hepatic antioxidant defence and reduction in serum enzymes of hepatic damage in rats fed with 2-acetylaminofluorene (AAF) [113]. Shamaan et al. [114] investigated the effect of tocotrienol on the activities of glutathione S-transferases (GSTs), glutathione reductase (GR) and glutathione peroxidase (GPx) in rats given 2-acetylaminofluorene (AAF) over a 20 week period. Liver and kidney GST and liver GR activities were significantly increased after AAF administration. Kidney GPx activities were significantly affected. In another experiment, alpha-tocopherol (αT) and gamma-tocotrienol (γT) were supplemented continuously for 8 weeks in the diets of normal rats and rats chemically induced with cancer using diethylnitrosamine (DEN), 2-acetylaminofluorene (AAF) and partial hepatectomy. Hepatocarcinogenesis was followed by determining the plasma gamma-glutamyl-transpeptidase (GGT) and alkaline phosphatase (ALP) activities as well as placental glutathione S-transferase (PGST) and GGT activities histochemically, at 4-week intervals. Male rats (*Rattus norvegicus*) were supplemented αT and γT at two different doses of 30 and 300 mg/kg diet. Elevation of plasma GGT activities and formation of PGST and GGT positive foci were attenuated significantly (P < 0.05) when αT and γT were supplemented simultaneously with cancer induction [115]. Ong et al. [116] investigated the effect of tocotrienol and tocopherol on glutathione S-transferase (GST) and gamma-glutamyltranspeptidase (GGT) activities in cultured rat hepatocytes. Tocotrienol and tocopherol significantly decreased GGT activities at 5 days in culture but tocotrienol also significantly decreased GGT activities at 1–2 days. Tocotrienol and tocopherol treatment significantly decreased GST activities at 3 days compared to the control but tocotrienol also decreased GST activities at 1–3 days. Tocotrienol showed a more pronounced effect at a dosage of greater than 50 microM tocotrienol at 1–3 days in culture compared to the control.

Another group of researchers investigated the effects of tocotrienol-rich fraction (TRF) on exercise endurance and oxidative stress in forced swimming rats. The results showed that the TRF-treated animals (268.0 ± 24.1 min for TRF-25 and 332.5 ± 24.3 min for TRF-50) swam significantly longer than the control (135.5 ± 32.9 min) and T-25-treated (154.1 ± 36.4 min) animals, whereas there was no difference in the performance between the T-25 and control groups [116]. The TRF-treated rats also showed significantly higher concentrations superoxide dismutase (SOD), catalase (CAT), and glutathione peroxidase (GPx), but lower levels in blood lactate, plasma and liver TBARS, and liver and muscle protein carbonyl. Their results suggested that TRF was able to improve the physiological condition and reduce the exercise-induced oxidative stress in forced swimming rats [117, 118].

### Anti-diabetic activity

For centuries, dietary antioxidants are well-known for the management of diabetes and some of them have been experimentally evaluated. However search for new anti-diabetic drugs continues. α-Tocotrienol (0.1 g/kg) significantly prevented oxidative damage in streptozotocin (STZ)-induced diabetic Osteogenic Disorder Shionogi (ODS) rat [119]. The TRF at a dose of 1 g/kg bodyweight significantly reduced streptozotocin induced diabetes in Sprague–Dawley rats [120]. It also effectively prevented increase in serum advanced glycosylation end-products (AGE) and malondialdehyde (MDA), and caused decrease in blood glucose and glycatedhemoglobin in diabetic rats. Intragastric administration of TRF from palm oil (200 mg/kg) significantly reduced the blood glucose level, oxidative stress markers and improved dyslipidemia in diabetic rats [121]. Diabetes is associated with a number of secondary complications such as neuropathy, retinopathy, nephropathy, lower limb amputations, etc. Kuhad et al. [122] evaluated the impact of tocotrienol on cognitive function and neuroinflammatory cascade in streptozotocin-induced diabetes. Streptozotocin-induced diabetic rats were treated with tocotrienol for 10 weeks. After 10 weeks of streptozotocin injection, the rats produced significant increase in transfer latency which was coupled with enhanced acetylcholinesterase activity, increased oxidative-nitrosative stress, TNF-alpha, IL-1beta, caspase-3 activity and active p65 subunit of NF-κB in different regions of diabetic rat brain. Co-administration of tocotrienol significantly prevented behavioural, biochemical and molecular changes associated with diabetes. Moreover, diabetic rats treated with insulin-tocotrienol combination produced more pronounced effect on molecular parameters as compared to their per se groups. Tocotrienol also prevented diabetic neuropathy in rat models [123, 124]. Oral administration of tocotrienol also significantly reduced the fasting serum glucose level in STZ induced diabetic rats by increased glucose metabolism and partly by hypotriglyceridemic effect of the plant extract. The extract also possessed oxidative stress reducing property in diabetic rats, which is believed to be a pathogenic factor in the development of diabetic complications [102, 123, 125–127]. The TRF from palm oil and rice bran oil was able to cause a significant reduction of elevated glucose-insulin index, signifying a potential insulin sensitizing effect in streptozotocin induced diabetic rats [127]. Oral administration of tocotrienol decreased the HbA1c, plasma glucose, lipids, peroxylipid (malonedialdehyde, MDA), albuminuria, proteinemia and uremia, and also improved the insulin sensitivity in various animal models [128–130]. It also prevents the incidence of long term complication in diabetic nephropathy [103, 119, 126, 127].

### Antihyperlipidemic activity

Hyperlipidaemia is a group of disorders in which a person has increased levels of lipids in the bloodstream. These lipids consist of cholesterol, phospholipids, triglycerides and cholestryl esters. Since lipids are insoluble in aqueous medium, they are usually carried in body fluids as soluble protein complexes called as lipoproteins. Hyperlipidaemia can lead to a number of metabolic diseases like cardiovascular dysfunction and atherosclerosis. Hyperlipidemia may also result from diseases such as diabetes, thyroid disease, renal disorders, obesity, alcohol consumption and liver disorders. Oxidative stress is regarded as an important risk factor for hyperlipidemia. Tocotrienols, because of their antioxidant activity, have long been used for reducing blood lipid levels. Tocotrienols from barley, oats, palm and rice bran have been demonstrated to lower cholesterol levels in animals and humans [59, 131–139], and that this effect has been reported to be mediated by suppressing HMG-CoA reductase activity through a post-translational mechanism [12, 134]. Magosso et al. [140] conducted a first clinical trial that demonstrated mixed palm tocotrienols exhibited significant hepatoprotective effects in hypercholesterolemic adults with non-alcoholic fatty liver disease, and this effect was proposed to be mediated by attenuating triglyceride accumulation via regulation of fatty acid synthase and carnitine palmitoyl transferase leading to a reduction of hepatic inflammation and ER stress [141, 142]. Furthermore, the study showed that tocotrienols significantly reduced serum levels of total cholesterol (TC), low density lipoproteins (LDL) and triglycerides (TG) compared to baseline. In patients with hyperlipidemia and carotid stenosis, long term treatment with palm oil (a rich source of tocotrienols) resulted in attenuation of oxidative modification of LDL and significantly prevented the initiation and propagation of atherosclerosis [143]. In two open clinical studies, tocotrienol (75 mg/day) supplementation for 2 months significantly reduced fasting blood lipid levels. TC levels dropped 13% and LDL-C dropped 9-15%, whereas high density lipoprotein-cholesterol (HDL-C) increased by 4-7%. In addition, δ-tocotrienol promoted metabolic health, where TG levels dropped 20-30% [144].

### Immunomodulatory activity

Tocotrienol’s virtues as an immune enhancer are only beginning to receive recognition in medicine. Tocotrienol enhanced both antigen specific (observed against humoral as well as Cell-mediated immune response) and nonspecific responses. Tocotrienols have been shown to induce favourable effects on the human immune system. A team of Malaysian scientists evaluated the effects of a tocotrienol compound on immune function, recruiting 108 healthy non-smoking women, ages 18 and 25 years, for a two-month long study. One group received 400 mg of tocotrienol compound per day, while the other group received placebo (400 mg/day soy oil) for the study period. Blood samples were analyzed at the start of the study, and again after 28 and 56 days. After 28 days of supplementation, all subjects received a single shot of tetanus toxoid (TT) vaccine. The team observed significant increases in levels of the anti-TT antibody, interferon (IFN)-gamma and interleukin (IL)-4 in the tocotrienol group, as compared with the placebo group. The researchers concluded that tocotrienols have immunostimulatory effects and potential clinical benefits to enhance immune response [145]. A study was conducted to determine the effect of dietary supplementation of tocotrienols on immune response of young and old C57BL/6 mice using a wide range of immune indices. The study demonstrated that dietary supplementation with T3 resulted in enhanced T cell proliferation [146]. In another study, the immunoregulatory effects of dietary α-tocopherol (Toc) and tocotrienols (T3) on humoral and cell-mediated immunity and cytokine productions were examined in Brown Norway rats. It was found that the IgA and IgG productivity of spleen and mesenteric lymph node (MLN) lymphocytes was significantly enhanced in the rats fed on Toc or T3, irrespective of concanavalin A (Con A) stimulation of the lymphocytes. On the contrary, the IgE productivity of lymphocytes from the rats fed on Toc or T-3 was less without ConA stimulation, but was greater in the presence of Con A, especially in the T3 group. Toc or T3 feeding significantly decreased the proportion of CD4^+^ T cells and the ratio of CD4^+^/CD8^+^in both spleen and MLN lymphocytes of the rats fed on Toc or T3. The interferon-γ productivity of MLN lymphocytes was higher in the rats fed on Toc or T3 than in those fed on a control diet in the presence of Con A, while that of spleen lymphocytes was lower in the rats fed on Toc or T3. In addition, T3 feeding decreased the productivity of tumor necrosis factor-α of spleen lymphocytes, while it enhanced the productivity of MLN lymphocytes. The results suggested that oral administration of Toc and T3 affected the proliferation and function of spleen and MLN lymphocytes [147]. The TRF was found to enhance immune response to tetanus toxoid (TT) immunisation in BALB/c mice. The production of anti-TT antibodies was augmented (P < 0.05) in mice that were fed with δ-T3 or TRF. The production of IFN-γ and IL-4 by splenocytes from the TRF treated mice was significantly (P < 0.05) higher. Production of TNF-α was also suppressed in the vitamin E supplemented mice [148]. All these findings suggest that tocotrienol could be a useful “natural complement” for immune boasting.

### Protection against cardiovascular disease

Cardiovascular disorder is the one of the most potent causes of death throughout the world and the role of tocotrienols in its prevention may have significant clinical implications. Out of a minimum of four different isoforms of tocotrienols, a- and c-tocotrienols are considered as the effective isoforms which possess the cardioprotective abilities. A number of studies have determined the cardioprotective abilities of tocotrienols and have been shown to possess novel hypocholesterolemic effects together with an ability to reduce the atherogenic apolipoprotein and lipoprotein plasma levels. In addition, tocotrienol has been suggested to have an antioxidant, anti-thrombotic, and antitumor effect indicating that tocotrienol may serve as an effective agent in the prevention and/or treatment of cardiovascular disease and cancer. The bioactivity exhibited is due to the structural characteristics of tocotrienols. Rich sources of tocotrienols which include rice bran, palm oil, and other edible oils exhibit protective effect against cardiovascular disorders [149].

Diseases that involve the heart and blood vessels remain the biggest cause of deaths worldwide. Coronary heart disease, cardiomyopathy, ischemic heart disease, heart failure, hypertensive heart disease, inflammatory heart disease and valvular heart disease are some of cardiovascular complications. Tocotrienol has long been used for various cardiac complications. Tocotrienols’ cardioprotective effects are mediated through their antioxidant mechanisms and their ability to suppress inflammation, and inhibit HMG-CoA reductase, a rate-limiting enzyme in cholesterol biosynthesis [11, 12, 150], and reduce the expression of adhesion molecules and monocyte–endothelial cell adhesion [151]. Tocotrienols were found to be more effective than α-tocopherol in depressing age-related increases in systolic blood pressure of spontaneously hypertensive rats [152]. TRF from rice bran oil improved lipid abnormalities, reduced the atherogenic index and suppressed the hyperinsulinemic response in rats with streptozotocin/nicotinamide-induced type 2 diabetes mellitus [153]. Tocotrienol significantly alleviated atherosclerotic iliac artery stenosis induced by both the endothelialization and high cholesterol diet. It also significantly lowered aortic contents of malondialdehyde and intimal thickening as well as preserved the internal elastic lamina in rabbits [154]. The tocotrienols also significantly alleviated the ischemia-reperfusion injury, and reduced infarct size in the ischemic region of myocardial tissue, through the downmodulation of c-Src and upregulation of phosphorylation of Akt, thus generating a survival signal [155]. Treatment of tocotrienols orally to pigs expressing hereditary hypercholesterolemia significantly reduced serum total cholesterol, LDL-cholesterol, apolipoprotein B, platelet factor 4, thromboxane B(2), glucose, triglycerides, and glucagon. The tocotrienols also lowered the hepatic HMG-CoA reductase activity and cholesterol and fatty acid levels in various tissues [156].

In one study, the effects of red palm oil on the myocardial nitric oxide-cGMP signaling pathway, associated with myocardial protection against ischemia, were investigated [157]. Treatment with red palm oil increased aortic output and increased levels of cGMP and polyunsaturated fatty acid in rat hearts suggesting that dietary red palm oil protects via the nitric oxide–cGMP pathway and/or changes in polyunsaturated fatty acid composition during ischemia/reperfusion. Newaz et al. [109] determined the effects of γ-tocotrienol on lipid peroxidation and total antioxidant status of spontaneously hypertensive rats. The γ-tocotrienol exhibited a dose dependent hypotensive effect on the systolic blood pressure of spontaneously hypersensitive rats. It also caused a significant drop in the mean arterial pressure in a dose dependent manner, decreased lipid peroxidation and increased the activity of antioxidant enzymes in hearts of rats. Myocardial ischemic injury results from severe impairment of coronary blood supply and produces a spectrum of clinical syndromes. In a study, γ-tocotrienol significantly reduced coronary perfusion pressure and heart rate. It exerted protection against myocardial injury by mitigating cardiac dysfunction and oxidative injury in rats and also by the differential interaction of MAPK with caveolin 1/3 in conjunction with proteasome stabilization, possibly by altering the availability of prosurvival and antisurvival proteins [158]. Diabetes mellitus is always accompanied by dyslipidemia, which is an important factor in the pathogenesis of diabetic complications, such as cardiovascular diseases and diabetic nephropathy. The TRF from palm oil and rice bran oil has been shown to decrease the serum lipid profile in type-1 and type-2 diabetic Wistar rats [127, 130]. It has also been reported to decrease the dyslipidemia induced diabetic nephropathy through the downregulation of the TGF-β expression [127].

### Neuroprotective effects

Various reports suggest that tocotrienols are neuroprotective [159–166]. Tocotrienols also have activity against Parkinson disease [167]. In one study, HT4 hippocampal neuronal cells were studied to compare the efficacy of tocopherols and tocotrienol to protect against glutamate-induced death. Tocotrienols were more effective than alpha-tocopherol in preventing glutamate-induced death. It was suggested that tocotrienols may have protected cells by an antioxidant-independent mechanism. Examination of signal transduction pathways revealed that protein tyrosine phosphorylation processes played a central role in the execution of death. Activation of pp60(c-Src) kinase and phosphorylation of ERK were observed in response to glutamate treatment. Nanomolar amounts of α-tocotrienol, but not α-tocopherol, blocked glutamate-induced death by suppressing glutamate-induced early activation of c-Src kinase. Overexpression of kinase-active c-Src sensitized cells to glutamate-induced death. Tocotrienol treatment prevented death of Src-overexpressing cells treated with glutamate [159].

A growing body of research supports that members of the vitamin E family are not redundant with respect to their biological function. Palm oil derived from *Elaeis guineensis* represents the richest source of the lesser characterized vitamin E, α-tocotrienol. One of 8 naturally occurring and chemically distinct vitamin E analogs, α-tocotrienol possesses unique biological activity that is independent of its potent antioxidant capacity. Current developments in α-tocotrienol research demonstrate neuroprotective properties for the lipid-soluble vitamin in brain tissue rich in polyunsaturated fatty acids (PUFAs). Arachidonic acid (AA), one of the most abundant PUFAs of the central nervous system, is highly susceptible to oxidative metabolism under pathologic conditions. Cleaved from the membrane phospholipid bilayer by cytosolic phospholipase A2, AA is metabolized by both enzymatic and nonenzymatic pathways. A number of neurodegenerative conditions in the human brain are associated with disturbed PUFA metabolism of AA, including acute ischemic stroke. Palm oil–derived α-tocotrienol at nanomolar concentrations has been shown to attenuate both enzymatic and nonenzymatic mediators of AA metabolism and neurodegeneration. On a concentration basis, this represents the most potent of all biological functions exhibited by any natural vitamin E molecule. Despite such therapeutic potential, the scientific literature on tocotrienols accounts for roughly 1% of the total literature on vitamin E, thus warranting further investigation [168].

A growing body of literature has begun to delineate the unique and potent biological properties of the natural vitamin E, αTCT [169]. To date, the neuroprotective qualities of αTCT in neurodegenerative disorders of the CNS are well characterized, with specific molecular targets (cPLA2, 12-LOX, and c-Src) and mechanisms of action identified. Beyond the CNS, αTCT has also demonstrated therapeutic promise in the treatment cancer and hypercholesterolemia. As a dietary source in humans, the oil palm represents the richest source of αTCT known today. Although tocotrienols are present in edible products such as palm oil, it remains questionable whether a dietary source alone could provide sufficient amounts of αTCT to humans [169], which is particularly relevant in diets that are typically devoid of palm oil and other natural sources of αTCT. Enrichment of αTCT from crude palm oil for dietary supplementation is achievable, and to date represents the most cost effective and readily available source of natural αTCT [168].

a-Tocotrienol (TCT) represents the most potent neuroprotective form of natural vitamin E that is generally recognized as a safe certified by the U.S. Food and Drug Administration. The recent work of Park et al. [170] addresses a novel molecular mechanism by which a-TCT may be protective against stroke in vivo. Elevation of intracellular oxidized glutathione (GSSG) triggers neural cell death. Multidrug resistance-associated protein 1 (MRP1), a key mediator of intracellular oxidized glutathione efflux from neural cells, may therefore possess neuroprotective functions. Stroke-dependent brain tissue damage was studied in MRP1-deficient mice and a-TCT-supplemented mice. Elevated MRP1 expression was observed in glutamate-challenged primary cortical neuronal cells and in stroke-affected brain tissue. MRP1-deficient mice displayed larger stroke-induced lesions, recognizing a protective role of MRP1. In vitro, protection against glutamate-induced neurotoxicity by a-TCT was attenuated under conditions of MRP1 knockdown; this suggests the role of MRP1 in a-TCT-dependent neuroprotection. In vivo studies demonstrated that oral supplementation of a-TCT protected against murine stroke. MRP1 expression was elevated in the stroke-affected cortical tissue of a-TCT-supplemented mice. Efforts to elucidate the underlying mechanism identified MRP1 as a target of a microRNA (miRNA). In a-TCT-supplemented mice, the miRNA was downregulated in stroke-affected brain tissue [170]. The work of Park et al. [170] recognizes MRP1 as a protective factor against stroke. Furthermore, findings of this study add a new dimension to the current understanding of the molecular bases of a-TCT neuroprotection in 2 ways: by identifying MRP1 as a a-TCT-sensitive target and by unveiling the general prospect that oral a-TCT may regulate miRNA expression in stroke-affected brain tissue [170]. Hence, the findings of their study add a new dimension to the current understanding of the molecular bases of a-TCT neuroprotection in 2 ways: by identifying MRP1 as a-TCT-sensitive target and by unveiling the general prospect that oral a-TCT may regulate microRNA expression in stroke-affected brain tissue. Neuroprotective, as well as hypocholesterolemic, properties of a-TCT make it a good candidate for nutrition based intervention in people at high risk for stroke. Transient ischemic attack, or mini-stroke, serves as a sentinel warning sign for high-risk stroke patients. Prophylactic stroke therapy therefore provides an opportunity for intervention in patients transient ischemic attack before a major stroke event. Outcomes of the current study warrant clinical assessment of a-TCT in transient ischemic attack patients. Furthermore, a-TCT is a nutrient that is certified by the U.S. Food and Drug Administration to be generally recognized as safe and is not a drug with potential side effects. Thus, a-TCT may be considered as a preventive nutritional countermeasure for people at high risk for stroke.

In order to determine whether the neuroprotective activity of alpha-tocotrienol is antioxidant-independent or -dependent, Khanna et al. [162] conducted a study using two different triggers of neurotoxicity, homocysteic acid (HCA) and linoleic acid. Both HCA and linoleic acid caused neurotoxicity with comparable features, such as increased ratio of oxidized to reduced glutathione GSSG/GSH, raised intracellular calcium concentration and compromised mitochondrial membrane potential. Mechanisms underlying HCA-induced neurodegeneration were comparable to those in the path implicated in glutamate-induced neurotoxicity. Inducible activation of c-Src and 12-lipoxygenase (12-Lox) represented early events in that pathway. Overexpression of active c-Src or 12-Lox sensitized cells to HCA-induced death. Nanomolar α-tocotrienol was protective. Knock-down of c-Src or 12-Lox attenuated HCA-induced neurotoxicity. Oxidative stress represented a late event in HCA-induced death. The observation that micromolar, but not nanomolar, α-tocotrienol functions as an antioxidant was verified in a model involving linoleic acid-induced oxidative stress and cell death. Oral supplementation of alpha-tocotrienol to humans results in a peak plasma concentration of 3 mM. Thus, oral α-tocotrienol may be neuroprotective by antioxidant-independent as well as antioxidant-dependent mechanisms [162]. In another study, Khanna et al. [164] tested the hypothesis that phospholipase A2 (PLA2) activity is sensitive to glutamate and mobilizes arachidonic acid (AA), a substrate for 12-lipoxygenase. Furthermore, the researchers examined whether α-tocotrienol (TCT) regulates glutamate-inducible PLA2 activity in neural cells. Glutamate challenge induced the release of [^3^H]AA from HT4 neural cells. Such response was attenuated by calcium chelators, ethylene glycol tetraacetic acid (EGTA) and 1,2-bis(o-aminophenoxy)ethane- N,N,N',N'-tetraacetic acid (BAPTA), cytosolic PLA2 (cPLA2)-specific inhibitor arachidonyltrifluoromethyl ketone (AACOCF3) as well as TCT at 250 nM. Glutamate also caused the elevation of free polyunsaturated fatty acid (AA and docosahexaenoic acid) levels and disappearance of phospholipid-esterified AA in neural cells. Furthermore, glutamate induced a time-dependent translocation and enhanced serine phosphorylation of cPLA2 in the cells. These effects of glutamate on fatty acid levels and on cPLA2 were significantly attenuated by TCT. The observations that AACOCF3, transient knock-down of cPLA2 as well as TCT significantly protected against the glutamate-induced death of neural cells implicate cPLA2 as a TCT-sensitive mediator of glutamate induced neural cell death. The study suggested that TCT provided neuroprotection through glutamate-induced changes in cPLA2. Tocotrienols have also been found to possess neuroprotective activity in animal models of diabetic neuropathy [103, 122, 124, 170] and alcoholic neuropathy [171, 172]. Tocotrienols have been reported to suppress the proinflammatory pathways in diabetes and chronic alcoholism, which in turn prevented the animals from cognitive impairment and oxidative-nitrosative stress.

### Positive effects on bone metabolism

**Figure 1:**
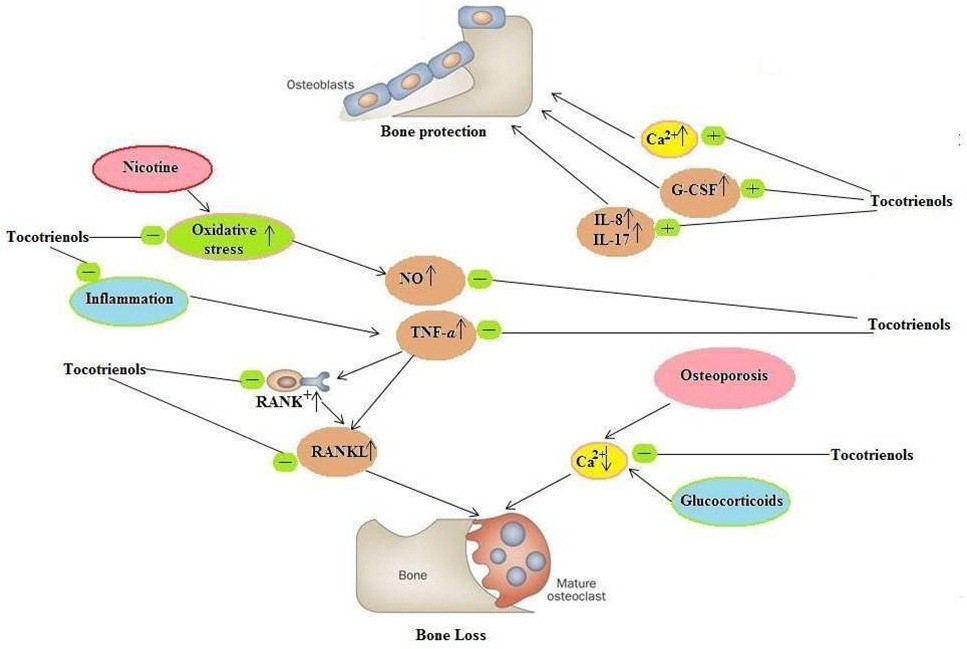
**Mechanistic action of tocotrienols in bone protection.** Tocotrienols prevent the increase in expression of TNF-α and nitric oxide (NO) due to nicotine administration, oxidative stress and inflammation and thus prevent osteoclast formation. Tocotrienols also downregulate the expression of Receptor activator of nuclear factor kappa-B (RANK) and Receptor activator of nuclear factor kappa-B ligand (RANKL). Osteoporosis and glucocorticoids also decrease the calcium ion concentration in bone leading to bone desorption. Tocotrienols prevent the desorption of calcium ions from bone, thus increasing the bone strength. Tocotrienols also increase the expression of interleukin-8 (IL-8), IL-17, granulocyte colony stimulating factor (G-CSF) which in turn lead to the formation of bone osteoblasts.

Bone is a specialised connective tissue hardened by mineralisation with calcium phosphate in the form hydroxyapatite ([Ca_3_(PO_4_)_2_]Ca(OH)_2_). Bone has well recognised mechanical functions: it provides rigidity and shape, protection and support for body structures, and aids locomotion. The rate of bone turnover, collagen matrix, size, structure, geometry and density all combine to determine the bone’s overall mechanical properties. Defects in these parameters will result in diseases such as osteoporosis, Paget’s disease of bone, osteoporosis and osteogenesis imperfecta. In order for the strength of the bone to be maintained, the process of bone turnover must be carefully regulated. Vitamin E and its various forms have been reported to help in the maintenance of bone metabolism [104, 105, 173–182]. Vitamin E supplements reversed nicotine-induced bone loss and stimulated bone formation [173, 174]. Tocotrienols are slightly superior to tocopherols in attenuating the effects of tobacco; γ-tocotrienol especially may have therapeutic potential to repair bone damage caused by chronic smoking. Other studies have shown that tocotrienols can reverse glucocorticoid-induced or free radical-induced bone loss in adrenalectomized rats [105, 175, 177] and improve normal bone structure [175, 177, 178] possibly through its antioxidant activity in bone [177, 182]. Ima-Nirwana et al. [180] showed that treatment with γ-tocotrienol (60 mg/kg body weight/day) reduced body fat mass and increased fourth lumbar vertebra bone calcium content in rats, while a-tocopherol was ineffective. Therefore, palm-oil derived γ-tocotrienol has the potential to be utilized as a prophylactic agent in prevention of the skeletal side effects of long-term glucocorticoid and tobacco use (Figure 1).

### Gastroprotective effects

Azlina et al. compared the impacts of tocopherols and tocotrienols on gastric acidity, gastric tissue content of parameters such as malondialdehyde and prostaglandin E2, and serum levels of gastrin and glucagon-like peptide-1 in rats exposed to restraint stress. They found that tocotrienol-treated animals, both stressed and non-stressed, had comparable gastric acidity and gastrin levels [183]. Both tocopherols and tocotrienols had gastroprotective effects against damage by free radicals generated in stress conditions, but only tocotrienols had the ability to block stress induced changes in gastric acidity and gastrin level. Another group showed that tocotrienols can prevent aspirin-induced gastric lesions through their ability to limit lipid peroxidation [184].

### Hepatoprotective activity

Liver the largest glandular organ of the body and the key organ of metabolism has a pivotal and immense task of detoxification of xenobiotics, environmental pollutants and chemotherapeutic agents. Hence this organ is subjected to a variety of diseases and disorders. In the absence of reliable hepatoprotective drugs in the allopathic (modern) medicinal system and the wide range of hepatic disorders, dietary antioxidants play an important role in the management of liver disorders.

Tocotrienol has been extensively studied for its efficacy against hepatic toxicity. Oral administration of tocotrienols offered a significant protection against 2-acetylaminofluorene (AAF) induced hepatotoxicity as assessed in terms of biochemical and histological parameters [113, 114]. Tocotrienols completely normalized the 2-acetylaminofluorene (AAF) induced increase in the levels of plasma and liver microsomal gamma-glutamyltranspeptidase (GGT) and liver microsomal UDP-glucuronyltransferase (UDP-GT) confirming *in vivo* hepatoprotective activity of tocotrienols against AAF induced toxicity. Tocopherol and γ-tocotrienol have been shown to prevent the nitrofurantoin induced damage in rat liver when administered for 10 weeks. The extract characteristically inhibited hepatic lipid peroxidation [111]. Tocotrienol also significantly increased the percent viability of cultured rat hepatocytes prepared from rats treated with diethylnitrosamine (DEN) and 2-acetylaminofluorene (AAF). Tocotrienol significantly decreased levels of glutathione S-transferase (GST) and gamma-glutamyltranspeptidase (GGT) activities in cultured rat hepatocytes at 1–3 days [116, 185]. In addition to their antioxidant activity, tocotrienols are found to increase the expression of drug metabolising enzymes such as cytochrome P450 enzyme (CYP450), UDP-glucuronosyltransferase 1A1 (UGT1A1) and multidrug resistance protein-1 (MDR1) via the activation of the pregnane-X-receptor (PXR) and steroid and xenobiotic receptor (SXR), which are the nuclear receptors that regulate drug clearance in the liver and intestine via induction of genes involved in drug and xenobiotic metabolism, thus increasing the activity of liver to metabolize the xenobiotics [186, 187]. Oral administration of TRF on exercise endurance and oxidative stress in forced swimming rats caused significant increase in the concentrations of liver glycogen, SOD, CAT, and GPx, as well as of muscle glycogen and SOD than the control and lowered levels of blood lactate, plasma and liver TBARS, and liver and muscle protein carbonyl. Taken together, these results suggest that TRF is able to improve the physiological condition and reduce the exercise-induced oxidative stress in forced swimming rats [108].

Organophosphorus insecticides (OPIs) may induce oxidative stress leading to generation of free radicals and alteration in antioxidant system of animals. Bhatti et al. [188, 189] conducted a study to investigate the possible protective role of vitamin E on ethion-induced hepatotoxicity in rats using qualitative, quantitative and biochemical approaches. The result of their study shows that in vivo administration of ethion caused a significant induction of oxidative damage in liver tissue as evidenced by increased level of LPO and decreased GSH content. Ethion toxicity also led to a significant increase in the activities of SOD, CAT, GPx and GST in liver tissue. In addition, decrease in GR activity was observed in ethion administered rats compared to control. Histopathological findings revealed that exposure to ethion caused damage in liver tissue. However, simultaneous supplementation with vitamin E restored these parameters partially. The results revealed that supplementation of vitamin E exhibited protective effect by inhibiting ethion-induced toxicity in liver and erythrocytes. Patel et al. [190] conducted a study to determine the concentrations of TE (200 mg mixed TE, b.i.d.) and TCP (200 mg alpha-TCP, b.i.d.) in vital tissues and organs of adults receiving oral supplementation. A total of eighty participants were studied. Skin and blood vitamin E concentrations were determined from healthy participants following 12 wk of oral supplementation of TE or TCP. Vital organ vitamin E levels were determined by HPLC in adipose, brain, cardiac muscle, and liver of surgical patients following oral TE or TCP supplementation (mean duration, 20 wk; range, 1–96 wk). Oral supplementation of TE significantly increased the TE tissue concentrations in blood, skin, adipose, brain, cardiac muscle, and liver over time. The alpha-TE was delivered to human brain at a concentration reported to be neuroprotective in experimental models of stroke. In prospective liver transplantation patients, oral TE lowered the model for end-stage liver disease (MELD) score in 50% of patients supplemented, whereas only 20% of TCP-supplemented patients demonstrated a reduction in MELD score. The results demonstrated that orally supplemented TE are transported to vital organs of adult humans. The findings of this study, in the context of the current literature, lay the foundation for Phase II clinical trials testing the efficacy of TE against stroke and end-stage liver disease in humans. All these findings are very promising and demand the attention of the scientific community for further exploration and evaluation of tocotrienols against hepatic toxicity.

### Nephroprotective activity

The kidneys are organs that serve several essential regulatory roles in vertebrate animals. They are essential in the urinary system and also serve homeostatic functions such as the regulation of electrolytes, maintenance of acid–base balance, and regulation of blood pressure (via maintaining salt and water balance). They serve the body as a natural filter of the blood, and remove wastes produced by metabolism in the form of urine. Just like liver, kidneys are also susceptible to a variety of diseases and disorders. Tocotrienols have been reported to possess significant nephroprotective activity. In one study, the effects of a long-term treatment with vitamin E (α-tocophenol), insulin, or their combination on renal damage in STZ-induced diabetic rats fed a high cholesterol diet was investigated. Increases in urinary albumin and lipid peroxide (LPO) excretions were observed in these diabetic rats, when both urinary parameters were measured at 8 and 15 weeks after STZ administration. Daily treatment with α-tocophenol, insulin, or their combination markedly suppressed the increase in the 24 h urinary albumin and lipid peroxide excretions. Furthermore, glycogen degeneration of distal tubules, fatty degeneration of glomerular endothelium and hypertrophy of glomeruli and mesangium were observed in the kidneys of the diabetic animals when histopathological evaluation was performed at 4, 8, and 15 weeks (glomerular and mesangial hypertrophy were observed only at 15 weeks). Combined α-tocophenol (vitamin E) and insulin treatment was the most effective at suppressing these renal histopathological changes. Hence, the results indicated that combined vitamin E and insulin treatment additively prevented the development and progression of renal damage in diabetic rats, possibly because of their antioxidant and hypolipidemic activity [191].

Tocotrienol (100 mg/kg) as well as TRF (200 mg/kg) from palm oil and rice bran oil prevents the kidneys from diabetic nephropathy in streptozotocin-induced type-1 and high-fat diet/streptozotocin induced type-2 diabetic rats. Diabetic rats produced significant alteration in renal function, increased oxidative-nitrosative stress, TNF-alpha, TGF-beta1, caspase-3 activity in cytoplasmic lysate and active p65 subunit of NF-κB in nuclear lysate of kidney of diabetic rats. Interestingly, administration of tocotrienol and TRF from rice bran oil and palm oil significantly and dose-dependently prevented biochemical and molecular changes associated with diabetes. Tocotrienols modulated the release of profibrotic cytokines, oxidative stress, ongoing chronic inflammation and apoptosis and thus exerts a marked renoprotective effect [103, 126, 127].

Tocotrienol also prevents the kidneys from ferric nitrilotriacetate (Fe-NTA) toxicity, a well-established nephrotoxic agent. Pretreatment with tocotrienol (50 mg/kg/day) for 7 days before Fe-NTA administration in rats significantly reduced the serum creatinine and BUN levels, reduced lipid peroxidation in a significant manner, and restored levels of reduced glutathione and superoxide dismutase. Tocotrienol pretreatment also attenuated the serum tumor necrosis factor-alpha levels and restored normal renal morphology [192]. TRF from palm oil (200 mg/kg, bw, orally, once daily for 21 days) was also found to prevent the kidneys in rats from potassium dichromate and Fenitrothion (FNT) induced acute renal toxicity [193, 194]. Nowak et al. [195] conducted a study to determine whether γ-tocotrienol (γT3) protects against mitochondrial dysfunction and renal proximal tubular cell (RPTC) injury caused by oxidants. Primary cultures of RPTCs were injured by using tert-butyl hydroperoxide (TBHP) in the absence and presence of γT3 or AT. ROS production increased 300% in TBHP-injured RPTCs. State 3 respiration, oligomycin-sensitive respiration, and respiratory control ratio (RCR) decreased 50, 63, and 47%, respectively. The number of RPTCs with polarized mitochondria decreased 54%. F(0)F(1)-ATPase activity and ATP content decreased 31 and 65%, respectively. Cell lysis increased from 3% in controls to 26 and 52% at 4 and 24 h, respectively, after TBHP exposure. γT3 blocked ROS production, ameliorated decreases in state 3 and oligomycin-sensitive respirations and F(0)F(1)-ATPase activity, and maintained RCR and mitochondrial membrane potential (DeltaPsi(m)) in injured RPTCs. GT3 maintained ATP content, blocked RPTC lysis at 4 h, and reduced it to 13% at 24 h after injury. Treatment with equivalent concentrations of AT did not block ROS production and cell lysis and moderately improved mitochondrial respiration and coupling. This is the first report demonstrating the protective effects of GT3 against RPTC injury by: i) decreasing production of ROS, ii) improving mitochondrial respiration, coupling, Delta-Psi (m), and F(0)F(1)-ATPase function, iii) maintaining ATP levels, and iv) preventing RPTC lysis. The data suggested that GT3 is superior to AT in protecting RPTCs against oxidant injury and may prove therapeutically valuable for preventing renal injury associated with oxidative stress.

### Radioprotective effects

Radiation-induced toxicity in various tissues is a manifestation of free radicals, oxidative stress, DNA damage [196], inflammation, [197] and apoptosis [198]. These different signaling pathways are known to have deleterious effects in various diseases such as hypertension, diabetes, and cancer progression [197, 199]. Various compounds such as antioxidants, thiols, antiapoptotic molecules, cytokines and growth factors have been tested against acute radiation injury [200–202]. Ghosh et al. [203] have shown that the prophylactic treatment with gamma-tocotrienol (GT3), 24 h prior to irradiation protects mice from radiation injury. Radioprotection by GT3 is associated with reduction of radiation-induced DNA damage [204] and inhibition of HMGCR-mediated-nitrosative stress [205]. GT3 is also shown to increase serum interleukin-6 (IL-6) and G-CSF levels which are known to stimulate hematopoiesis. Induction of these cytokines may contribute to radioprotective action of GT3 [206].

The radioprotective effect of GT3 depends not only on its antioxidative properties but also on its abilities to concentrate in endothelial cells and inhibit the enzyme, 3-hydroxy-3-methylglutaryl-coenzyme A (HMG-CoA) reductase. HMG-CoA reductase inhibitors are commonly used in the treatment of hyperlipidemia disorders but in addition have a plethora of vasculoprotective, anti-inflammatory and anti-fibrotic effects mediated by endothelial nitric oxide synthase (eNOS, NOS3) [207, 208].

In an attempt to enhance the radioprotective efficacy of GT3, Kulkarni et al. [209] tested the effect of PTX, a methyl derivative of xanthine, in combination with GT3. They have shown that the increase in the radioprotective efficacy of GT3 by combining it with pentoxifylline (PTX) was due to PDE inhibition, an effect that was reversed by calmodulin administration [209]. PTX has similar antioxidant, vasculoprotective, anti-inflammatory and anti-fibrotic properties and similarly increases eNOS activity through an increase in intracellular cyclic adenosine monophosphate (cAMP) [210–212].

PTX is an FDA-approved non-specific PDE inhibitor used for intermittent claudication [213, 214]. PTX has been used alone and in combination with vitamin E (alpha-tocopherol) in preclinical and clinical studies to reduce long-term effects of radiation such as fibrosis [215–217]. Beneficial effects of PTX are contributed to its ability to inhibit proinflammatory cytokine signaling such as tumor necrosis factor-alpha (TNF-α) accumulation [218]. According to these studies, there was significant reduction in TNF-α in presence of PTX in early (2 weeks) as well as late (24 weeks) phase of radiation injury. It was recently shown that combining PTX with GT3 increased the radioprotective efficacy of GT3 in protecting mice from acute radiation injury [218]. These studies indicated that even though PTX increased the radioprotection in mice treated with GT3, its mechanism of protection was independent of endothelial nitric oxide synthase (eNOS). PTX was shown to increase nitric oxide production [210] by increasing cAMP levels.

Berbee et al. [219] examined the effects of GT3 in combination with PTX on total body irradiation (TBI)-induced acute hematopoietic, intestinal and vascular injury and subsequent mortality. They used eNOS-deficient mice to determine whether protection against lethality from either drug alone or the combination required the presence of eNOS. Combined therapy was significantly more effective in improving postirradiation survival than treatment with GT3 only, but the effect on postirradiation lethality did not require the presence of eNOS. Moreover, their data suggested that administration of GT3 together with PTX may modulate the hematopoietic radiation response by the induction of hematopoietic stimuli. GT3 combined with PTX also reduced postirradiation intestinal injury and vascular oxidative stress compared to vehicle, but no additional benefit was observed by the addition of PTX to GT3 compared to treatment with GT3 alone [219].

## Toxicity and dosage

To start with, quoting Paracelsus (1493–1541 Switzerland), “in all things there is a poison, and there is nothing without a poison. It depends on only upon the dose whether a poison is a poison or not”. Under dietary considerations, tocotrienol has been regarded as a safe biomolecule and experimental studies have also supported this view. There is no possible report of any adverse reactions caused by tocotrienol, except that in a study done on experimental animals in a 13-week oral toxicity study performed in Fischer 344 rats of both sexes at dose levels of 0% (group 1), 0.19% (group 2), 0.75% (group 3) and 3% (group 4) of a diet preparation in powdered form. On hematological examination, significant decrease in mean corpuscular volume (MCV) was observed in all treated males. Platelets were significantly reduced in group 3 and 4 males. Hemoglobin concentration, MCV, mean corpuscular hemoglobin and mean corpuscular hemoglobin concentration were significantly decreased in group 3 and 4 females and hematocrit in group 4 females. On biochemical examination, increase in the albumin/globulin ratio (A/G) and alkaline phosphatase in all treated males, elevated alanine transaminase in group 4 of both sexes and increases in asparagine transaminase and gamma-glutamyl transaminase in group 4 females were observed. With regard to relative organ weights, liver weights in group 4 of both sexes and adrenal weights in all treated males demonstrated an increase, and ovary and uterus weights in group 4 females were reduced. A slight hepatocellular hypertrophy in group 3 and 4 males, and reduction of cytoplasmic vacuolation in the adrenal cortical region in group 4 males were observed. Because of pathological changes in male liver and hematological changes in females, the no-observed-adverse-effect level (NOAEL) was concluded to be 0.19% in the diet (120 mg/kg for male rats and 130 mg/kg body weight/day for female rats) [220]. Since, most of the studies have been in favor of tocotrienols, however, it needs to be replicated in human populations to evaluate the safety and efficacy of tocotrienols as a therapeutic agent or drug.

There are convincing evidence that tocotrienols are detectable at appreciable levels in the plasma after short term and long term supplementations. However, there is insufficient data on the range of plasma concentrations of tocotrienols that are adequate to demonstrate significant physiological effects. Although the pharmacokinetics of tocotrienols are distinctly different from tocopherols which are well studied and remained longer in blood circulation, biodistribution study showed considerable accumulation of tocotrienols in vital organs. In the perspective of therapeutic efficacy, it is evident that the outcome of clinical evaluations is not only affected by the bioavailability of tocotrienols. In view of the limited understanding, more studies on the mechanisms of absorption are essential [221].

In an effort to determine the therapeutic window for tocotrienols, a number of long term clinical studies have been carried out using TRF and tocotrienol derivatives. The majority of these trials were focused on lipid profile as tocotrienols were found to inhibit HMG-CoA reductase [76–78]. However, the optimum dosing regimen to induce therapeutic effects remained unclear.

Although most studies were conducted in rodents and animals, they serve as a basis for clinical evaluations to establish their health benefits in humans. A number of clinical trials were conducted to examine the multi-faceted health benefits of tocotrienols in different populations. The bioavailability and efficacy of TRF may vary in different populations. tocotrienols seem to respond differently to a range of age groups but did not show consistent efficacies in the target study populations. Most of the studies conducted in patients with chronic diseases had relatively small sample size. This demonstrates the need to conduct randomized controlled trials in larger population to confidently evaluate the therapeutic potentials of tocotrienols [221].

Patel et al. [190] have determined the concentrations of TE (200 mg mixed TE, b.i.d.) and TCP [200 mg a-TCP, b.i.d.)] in vital tissues and organs of adults receiving oral supplementation. Skin and serum vitamin E concentrations were determined from healthy participants following 12 wk of oral supplementation of TE or TCP. The vitamin E levels in vital organ were determined by HPLC in adipose, brain, cardiac muscle, and liver of surgical patients following oral TE or TCP supplementation (mean duration, 20 wk; range, 1–96 wk). Oral supplementation of TE significantly increased the TE tissue concentrations in blood, skin, adipose, brain, cardiac muscle, and liver over time. a-TE was delivered to human brain at a concentration reported to be neuroprotective in experimental models of stroke. The finding of the study is the foundation for Phase II clinical trials testing the efficacy of TE against stroke and end-stage liver disease in humans [190]. Their work provides the first evidence on tissue availability of TE in vital organs of adult humans following oral supplementation to characterize multiple vital organ concentration of TCP in adults. TE was delivered and accumulated in vital human organs supports future studies to identify specific mechanisms of tissue delivery and metabolism. The outcomes of this work provide clear evidence that oral TE supplementation enriches its concentration in whole blood, adipose, skin, brain, cardiac muscle, and liver [190].

## Conclusion

In recent years, the basic research on vitamin E has expanded from primarily focusing on αT and its antioxidant effects to investigation of different tocopherols and tocotrienols, their metabolism, and their non-antioxidant activities including anti-inflammatory properties. Despite well-documented beneficial effects [3, 220], as well as negative association between αT intake and chronic diseases, supplementation with αT has failed to offer consistent benefits for the prevention of chronic diseases, including cancer and cardiovascular diseases, in many large clinical intervention studies [4, 5, 222, 223]. αT may be beneficial to individuals with deficiency of αT and/or other micronutrients [220], that can be caused by low dietary intake of this vitamin E or depletion of αT due to pathological condition or malnutrition associated with smoking, alcoholism, and malabsorption. Under these subclinical conditions, αT supplementation is likely to be beneficial, as indicated in the Linxian study in a population with deficiencies of micronutrients [224] and the ATBC study including heavy smokers [225]. On the other hand, αT supplementation did not show beneficial effects in people with adequate nutrient status [4, 5].

In contrast to αT, despite no evidence that deficiency of other vitamin E forms would result in obvious clinical symptoms, accumulating evidence suggests that γT, δT, and tocotrienols seem to have unique properties that are superior to αT and relevant to prevention and therapy against chronic diseases even under conditions with adequate αT status. It is noteworthy that these bioactivities of tocopherols and tocotrienols including anti-inflammatory properties have been identified by mechanistic studies and subsequently substantiated in some preclinical models as well as clinical studies [44].

Hence, tocotrienols possess neuroprotective, antioxidant, anti-cancer and cholesterol lowering properties. Tocotrienols are thought to have more potent antioxidant and free radical scavenging properties due to their better distribution in the lipid layers of the cell membrane. In spite of the promising potential, the experimental analysis of tocotrienols accounts for only a small portion of vitamin E research. However, recent studies have enforced a serious reconsideration of this conventional perception and this review article will go a long way in reiterating research pursuit.

## Authors’ original submitted files for images

Below are the links to the authors’ original submitted files for images. Authors’ original file for figure 1
