# Supplementary figures and images for: Pharmacological potential of tocotrienols: a review
Source: Nutr Metab (Lond). 2014 Nov 12;11:52. doi: 10.1186/1743-7075-11-52 (PMC4247006; doi:10.1186/1743-7075-11-52)

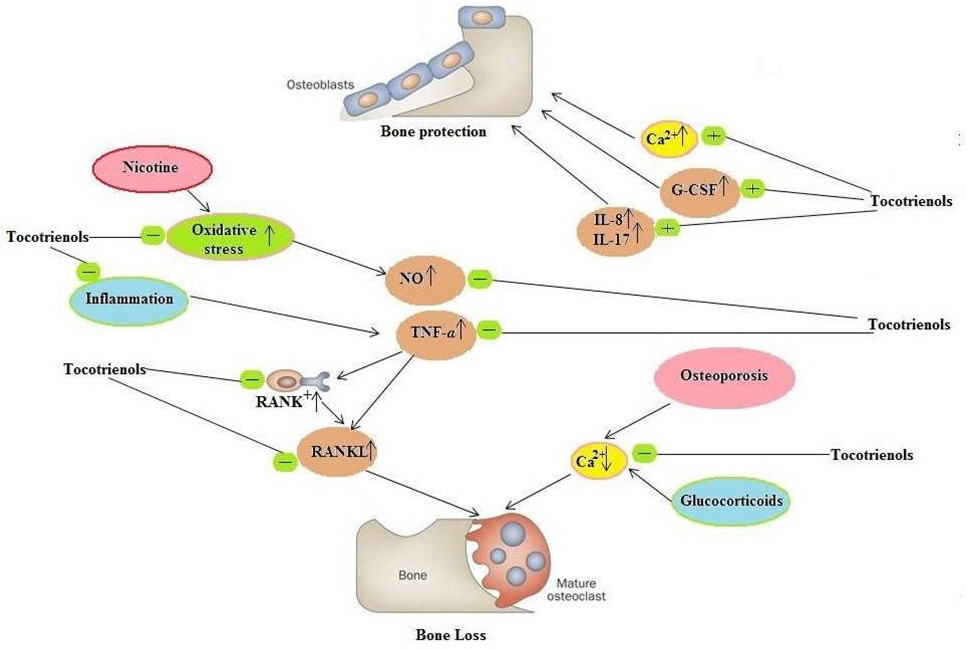

Supplement: Supplementary file 1 — Authors’ original file for figure 1 [file 12986_2014_623_MOESM1_ESM.jpg]
